# New skin for the old RNA-Seq ceremony: the age of single-cell multi-omics

**DOI:** 10.1186/s13059-017-1300-5

**Published:** 2017-08-24

**Authors:** Maayan Baron, Itai Yanai

**Affiliations:** Institute for Computational Medicine, NYU School of Medicine, 430 East 29th St., New York, NY 10016 USA

## Abstract

New methods for simultaneously quantifying protein and gene expression at the single-cell level have the power to identify cell types and to classify cell populations.

While we are taught to never judge a book by its cover, covers may actually be revealing. In the case of a cell, the surface proteins on its “cover” are unique to particular cell types: for example, CD3 for T cells and CD19 for B cells. With such markers in hand, populations of cells can be classified into the cell types they contain, in particular using fluorescence-activated cell sorting (FACS) analysis with a panel of antibodies. Over the past 5 years, however, a newer technology for characterizing populations has emerged, known as single-cell RNA-seq. Similarly to FACS, cells can be clustered according to their transcriptomes and cell types and subpopulations become readily identifiable [1, 2]. For example, when we previously studied pancreatic tissue in mouse and human we identified 15 cell types and subpopulations of the ductal cells [3]. However, it was not known if the cell-surface markers and transcriptomes provided consistent information. What would the other measurable quantities reveal? Ultimately, it appears that many further insights could be gleaned from an analysis of cells with several high-throughput methods, all at once. We would want to integrate RNA-seq measurements with genome sequencing, protein profiles, post-transcriptional regulation, metabolomics and lipidomics, together with the cellular localization of each—all at single-cell resolution, of course. Such complete characterization of cells at the population level would be a true treasure trove for insights into cellular physiology and pathological states.

A recent work published in *Nature Methods* has made a significant step forward toward multi-omics [4] by producing both transcriptomes and cell-surface protein quantifications on populations of cells.

## Cytometry by sequencing

**Fig. 1:**
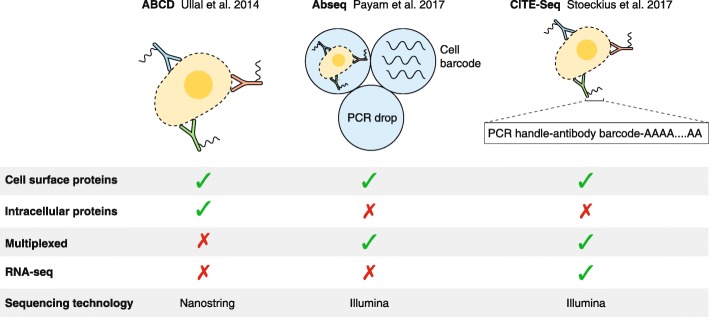
New methods for single-cell protein profiling. In the antibody barcoding with photocleavable DNA platform (ABCD) platform, cells are permeabilized and stained using a panel of antibodies. The tagged DNA is cleaved, amplified by PCR, and sequenced using Nanostring technology. The Abseq method is performed by encapsulating stained cells, tagging each cell with a unique barcode, and PCR amplifying and sequencing using Illumina technology. The cellular indexing of transcriptomes and epitopes by sequencing (CITE-Seq) method utilizes poly(A) oligonucleotides to profile cell-surface proteins and it can be coupled with single-cell RNA-seq protocols such as Drop-Seq and Chromium (10×)

The method—called CITE-Seq (cellular indexing of transcriptomes and epitopes by sequencing)—can be seen as a composite of two main concepts for how to derive cell-surface proteomics and transcriptomics from individual cells: DNA-conjugated antibodies and single-cell RNA-seq [4]. Detecting protein levels in individual cells is challenging as a result of low starting amounts and the lack of direct amplification methods common for nucleic acids. New techniques for protein profiling have been published in 2014 and earlier this year [5, 6]. The main insight for how to derive cell-surface proteomics is to tag proteins with antibodies conjugated to oligonucleotides (Fig. 1). By converting the detection of a protein to an oligonucleotide, the signal can then be amplified by exploiting Watson–Crick pairing of nucleic acids. This notion has been called “cytometry by sequencing” [4]. The identity of each protein is encoded in the oligonucleotides, which recapitulate a large number of distinguishable proteins: a sequence of length *N* corresponds to 4^*N*^ unique sequences, and therefore even a sequence of eight bases would theoretically suffice for capturing all cellular proteins.

Ullal et al. [5] first demonstrated this approach by developing the antibody barcoding with photocleavable DNA platform (ABCD; Fig. 1). The authors processed bulk samples of approximately 100 cells each, as well as samples containing individual cells, from a fine-needle aspiration and profiled over 90 proteins spanning cancer-relevant pathways. The cells are permeabilized before staining, thus enabling characterization of surface and intracellular proteins. In particular, in bypassing FACS and using only non-specialized instruments, ABCD is attractive for many clinical applications. The main constraint, however, is the lack of multiplexing of the different samples/cells, which thereby limits the handling to only a few samples at a time. More recently, the Abseq method was introduced, which utilizes custom microfluidics devices [6] to achieve a multiplexed version of cytometry by sequencing (Fig. 1). First, cells are incubated with a variety of antibodies conjugated with oligonucleotides encoding the protein identity, followed by encapsulation in drops and pairing with additional oligonucleotides to barcode the cells. Altogether, this method requires three separate microfluidic chips, and constitutes an impressive technical feat for single-cell proteomics.

## RNA-Seq and cell-surface proteomics in a drop

As in Abseq, cells in the CITE-Seq method are first incubated with cell-surface antibodies conjugated with oligonucleotides encoding the protein identity. CITE-Seq’s second underlying concept is the application of single-cell RNA-seq. The particular novelty is the design of the oligonucleotides tagged to the antibodies which contain a poly(A) region compatible with existing single-cell RNA-seq methods, either well-based or droplet-based. To demonstrate its general applicability, Stoeckius et al. [4] successfully implemented CITE-Seq with two established high-throughput methods—Drop-Seq and 10 × —to profile in parallel both the transcriptome and several cell-surface proteins of immune cells.

Using an antibody for CD8 as a proof-of-principle, Stoeckius et al. [4] demonstrated that the expression profile is comparable with the results detected by FACS. This is an important comparison since FACS has been the gold standard for the past two decades for profiling protein levels in millions of cells, for its speed, sensitivity, and capacity to profile tens of proteins at once (or even more if using multiplexing approaches) [7]. Extending to a broader set of ten immune cell surface proteins, Stoeckius et al. [4] showed that identifying cell types using protein expression profiles generally corresponded to RNA expression. This marks a multi-omics first in quantifying both surface proteins and transcriptomes in a population of cells. Surprisingly, the correlations between mRNA and protein levels were low for individual cells (0.02 < *R* < 0.53), though higher when averaging across cell types (0.58 < *R* < 0.95). Moreover, the authors showed that using CITE-Seq enhanced the characterization of known subtypes of natural killer cells (CD56 bright and dim), which was not previously detected using single-cell RNA-seq methods. Thus, with the combination of both surface proteins and transcriptomics, new subpopulations may be revealed which would not be possible without their combination.

## Future directions

Recently, the construction of a human cell atlas through a large-scale collaborative project has been proposed [8]. While single-cell RNA-seq is currently best positioned to provide the methodology for such an atlas, CITE-Seq now provides a possible additional layer of information. By classifying cells based upon a multi-omics approach, a refined atlas may be possible. How might CITE-Seq be scaled-up to provide a richer proteome beyond cell-surface proteins? A method for mild permeabilization seems a necessary addition to the protocol in order to also capture intracellular proteins. Practical issues regarding the number of antibodies used in parallel would also need to be addressed, in particular the issue of cross-reaction with unwanted epitopes. In addition, scaling-up to all proteins in a cell may introduce biases based upon differences in antibody affinities. Overall, these are exciting times in which both the cover and the inside of the cell are used to characterize its biology.

## Abbreviations

ABCD: Antibody barcoding with photocleavable DNA platform
CITE-Seq: Cellular indexing of transcriptomes and epitopes by sequencing
FACS: Fluorescence-activated cell sorting
